# The Book World of Medicine and Science

**Published:** 1899-04-22

**Authors:** 


### The Book World of Medicine and Science.

The Cry of the Children in the Twentieth Century; or, The Anti-Vaccinationists. By Mrs. Bray. (London: The Sanitary Publishing Company. Price 1d., or 6s. per 100.)

There is nothing like a little tale for impressing a simple fact upon a simple mind, and when writing such a tale one must take care to write it strong. Too much wrapping up is a mistake where one wants to point a moral, especially when both tale and moral have to be got in for a penny, and there can be no doubt that Mrs. Bray has avoided any error of the sort. The tale is that of a family of unvaccinated children in a village in which small-pox broke out, the father being a leading spirit among the anti-vaccinationists. But there were a pair of twins, the mother's last possession; and these she secretly got vaccinated. As the result of the outbreak one child after another was attacked. Some of them died, others came back from the hospital. "Was that Eva? Was that the brilliant, lovely girl who went away? Those thickened features, that coarse skin, those indelible marks all over the face, those shrunken eyes? And her father, looking on her, knew that her beauty was gone for ever, and almost wished her in her grave. And Willie? Well, Willie was blind for life." The vaccinated twins, however, were safe. "It was over at last, and the village had buried its dead. . . . And the 'cry of the children' rang right through England; and the cry rang out for vengeance against the anti-vaccinationists." The colouring is vigorous, but the moral is correct, and as for the facts, they, more's the pity, are true enough. A book like this will do more good than many handbills.

The Medical Annual and Practitioner's Index. 1899. Seventeenth year. (Bristol: John Wright and Co. Price 7s. 6d. net, post free.)

This volume fully maintains the reputation which the Medical Annual has earned for itself as a reliable and complete presentment, not merely of what is new in the various departments of practice, but of the present state of our knowledge in regard to the subjects dealt with. Many workers, well known in their various departments, have combined to produce this result, and it speaks well for the editing to which their contributions have been subjected that there is so little overlapping of the different papers. In unity and completeness the work is a great advance over what it was some years ago, which no doubt is largely due to practice in collaboration between editors and contributors. Among the special articles to which attention may be specially directed by reason of their length or their importance, may be mentioned "Practical X-Ray Work,' by Norris Woolfenden; "Fibromata," by Colcott Fox, with a couple of most remarkable photographic illustrations; "Gleet," and other articles on the surgery of the urinary organs, by Hurry Fenwick, with coloured illustrations of the appearances seen by the urethroscope; "Diseases of the Heart," by Alfred Carter, a most interesting *résumé* of the present position; a series of articles on abdominal and intestinal surgery, by Priestley Leech, beautifully illustrated; "Mastoid Operations," a well-illustrated and practical article; "Phthisis," "Thorax," "Climatic Treatment," and other articles on pulmonary diseases, by F. de Havilland Hall; "Skull Surgery," by Seneca D. Powell, of New York, illustrating the use of the electric saw; and the continuation of the "Atlas of Bacteria Pathogenic in the Human Species," which was begun last year, by Samuel G. Shattock. Among the miscellaneous articles we would draw attention to the record of legal decisions affecting medical men and the public health. Altogether, it is a most useful volume, and is excellently got up. The many coloured illustrations form a marked feature of the book.

Parish Problems; or, a Word with Everybody about the Parish Councils Act. By Lady Baker. (London and Westminster: Gardner, Darton, and Co.)

There are many people who find the study of Acts of Parliament very dull work, indeed there are not a few who, however much they may wish for information on such subjects, entirely decline to study the authorities at first hand. To such Lady Baker's little book will be a comfort, as it weaves into the conversations which take place in the course of a little story a description of many of the provisions of the Parish Councils Act. It is a somewhat curious omission that nowhere, so far as we have seen, is the full title of the Act mentioned; even the date at which it became law does not seem to have been referred to. That, however, is perhaps a matter of small importance, so far as the class of readers aimed at is concerned. Evidently, Lady Baker has taken much pains in studying the Act, and the ingenuity with which she has induced the various characters in the play to discuss its many-sided bearings deserves the highest praise.

Harry Ingleby, Surgeon. By F. Y. Webb. (London: Fisher Unwin. 6s.)

The author of Harry Ingleby has depicted the character of a young medical man struggling against the difficulties of his career in the sordid surroundings of a slum practice. The character is not a strong one, but it is one full of humanness in its patient persevering, its self-renunciation, and its sympathy with the suffering. The author has evidently much sympathy with child life, and one of the most touching descriptions in the book is that of a dying child whose life flame is kept flickering to its close by its desire "to help mother." The most interesting part of the story is that connected with Ingleby's student days, and later on with life amongst his patients. His medical "experiences" and the scenes in hospital should interest those who know something of this phase of existence.

Hovis Cycle Road Map (Publisl ed by the Hovis Bread Company).—The Hovis Bread Company are issuing a series of good road maps for cyclists, with a list of hotels and houses where accommodation may be had at moderate price. The price of the map in linen cover is 6d.

